# Bridging Psychology and Mathematics: Can the Brain Understand the Brain?

**DOI:** 10.1371/journal.pbio.0020297

**Published:** 2004-09-14

**Authors:** Mariano Sigman

## Abstract

Mathematical measures of complexity shed light on why some concepts are inherently more difficult to learn than others

*“It was not only difficult for him to understand that the generic term dog embraced so many unlike specimens of differing sizes and different forms; he was disturbed by the fact that a dog at three-fourteen (seen in profile) should have the same name as the dog at three-fifteen (seen from the front).…Without effort, he had learned English, French, Portuguese, Latin. I suspect, nevertheless, that he was not very capable of thought. To think is to forget a difference, to generalize, to abstract. In the overly replete world of Funes there were nothing but details, almost contiguous details.” —Jorge Luis Borges, “Funes the Memorius”*

We are told scientists are divided into experimentalists and theoreticians. The dialectic description of the dynamics of science, with one tribe gathering data and collecting evidence and another tribe providing form to these observations, has striking examples that argue for the importance of synthesis. The 16th century revolution, which settled the way in which we see the sky today, is probably one of the best examples of how comparatively ineffective each of these tribes can be in isolation. Tycho Brahe, the exquisite observer, who built, calibrated, and refined instruments to see in the sky what no one else could, collected the evidence to prove a theory that Copernicus had already stated years before (in a book he dedicated to the Pope). It was only many years later that Galileo established the bridge between theory and observation; he understood the data in terms of the theory and thereby cemented the revolution. Copernicus's statements, showed Galileo, were not only figments of his imagination; they were an adequate description of the universe as he and Brahe had observed it.

Since my first steps in biology, after a prompt departure from physics and mathematics, I have looked for such encounters between theory and experiment. I began studying the visual system and the series of fundamental works by Atneave (1954), Barlow (1960), and Atick (1992) on the relationship between our visual world and the way the brain processes it. Their research was based on a simple hypothesis: (1) the images that we see are highly redundant and (2) the optic nerve is a limited channel, thus the retina has to compress information. Compression, redundancy? How do such concepts relate to the biology of the brain?

In the middle of the last century, working on the problem of communications, languages, codes, and channels, Claude Shannon proposed a very elegant theory that formalized intuitive ideas on the essence (and the limits) of communications (Weaver and Shannon 1949). One of its key aspects was that, depending on the code and on the input, channels are not used optimally and thus do not carry all the information they potentially could. When we compress (zip) a file, we are actually rewriting it in a more efficient (though not necessarily more intelligible) language in which we can store the same amount of information in less space. This compression has, of course, a limit (we cannot convey the universe in a single point), and the mere existence of an optimal code is central to Shannon's idea. Attneave was probably the first to think that these ideas could help unravel how the brain worked, and in a long series of works relating these ideas to experimental data, it was shown that the retina's business is mainly to get rid of the redundancies in the visual world.

About four years ago, Jacob Feldman published a paper, similar in spirit, proposing a simple explanation for a long-standing problem in cognitive science about why some concepts are inherently more difficult to learn than others (Feldman 2000). An article whose first reference is to work carried out 50 years previously makes us suspect that an important gap is being filled. As in the previous experiments, Feldman borrowed a theory—he did not invent it—to explain longstanding and previously unexplained research in this area. Feldman's idea was based on a theory developed by Kolmogorov that established formal mathematical grounds to define and measure complexity. Kolmogorov's theory was focused on categories, which are just subsets of a universe, a bunch of exemplars that constitute a group. “Dogs” is a category in the universe of animals. Different statements can define the same category, for example, “big dogs” and “dogs that are not small” are the same group, and some information in the statement may be irrelevant because it does not aid in determining which elements belong or not. In the same way that Shannon spoke of a non-redundant code, Kolmogorov showed that categories could be defined with an optimal (non-redundant) statement. The length of this statement defines a measure of complexity termed Kolmogorov complexity.

To visualize the intuitive nature of his theory it helps to do a thought experiment. Imagine a set of objects defined by, say, three features: form, color, and size. And imagine, for simplicity, that each feature is binary, that is, there are only two possible cases. Size is big or small, color is yellow or red, and shape can be either a triangle or a circle. This defines, of course, a universe of eight objects. We can now define categories within this universe: for example, all the circles (a category of four exemplars), or all the big and yellow objects (two exemplars), or all the triangles that are not red (again two) (see Figure 1). We can also define a category by enumeration, for example, the small red triangle, the big yellow circle, and the small yellow circle (three exemplars). Some rules (and thus the groups defined by these rules) are intuitively easier to define than others. “All the circles” is an easier statement to make (and probably to remember) than “small circles and yellow big objects.” This notion of difficulty is what Kolmogorov's theory formalized, stating that complexity was the length of the shortest definition (among all the possible definitions) of a given set. From this thought experiment, we can understand the logic of Feldman's paper, which showed that. Kolmogrov complexity is very closely related to our intuitive notion of conceptual difficulty. Feldman presented subjects with all possible categories (of a fixed number of exemplars) in different universes and showed that the critical parameter to rank the difficulty of a given subset was its Kolmogrov complexity. Moreover by explicitly presenting all the members and the nonmembers of a category to naïve subjects, he showed that we can spontaneously reduce a category to its minimal form and remember it without any explicit instruction. Thus, what Feldman found, following the original ideas of Shepard, was that our psychological measure of complexity—our difficulty in defining and remembering a category or concept—is also determined by the Kolmogorov complexity that describes it.[Fig pbio-0020297-g001]

**Figure 1 pbio-0020297-g001:**
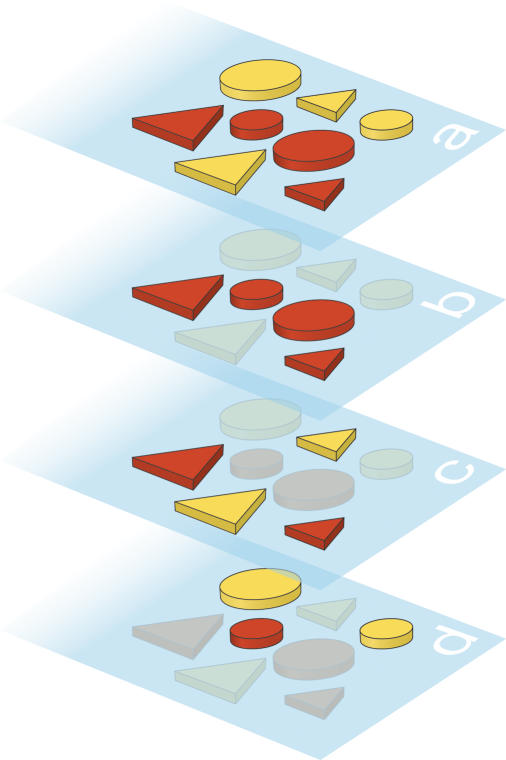
Visualizing Kolmogorov´s Complexity Intuitive categories can be defined by short statements. The universe: circles and triangles, red and yellow, big and small (A). Examples of easy categories: red objects (B); triangles (C). Example of a difficult category: yellow circles and small red circles (D).

This essay is, in a way, about how we avoid becoming Borges's character Funes, who could not understand repeated observations as exemplars of a common rule and thus could not synthesize and categorize. Simply, he could not think. Probably the most disappointing moment of Feldman's paper comes at the very end, where it deals with its (somehow unavoidable) recursive quest. Understanding why some concepts are difficult to learn may itself be difficult to learn. Modern mathematics, together with Kolmogorov complexity and information theory, has taught us another fundamental concept that may be relevant when trying to understand the logic of the mind. In a long series of paradoxes enumerated by Bertrand Russell, Kurt Goedel, and others, we learn that a formal system that looks at itself is bound to fail. At the very end of his paper, Feldman writes, “In a sense, this final conclusion [that psychological complexity is Boolean complexity] may seem negative: human conceptual difficulty reflects intrinsic mathematical complexity after all, rather than some idiosyncratic and uniquely human bias.” Who invented mathematics? The Martians? On the contrary, I believe this result supports a more naturalistic and less Platonic conception of mathematics. Formal ideas in mathematics are not arbitrary constructions of an arbitrary architecture; rather, they reflect the workings of the brain like a massive collective cognitive experiment. Mathematics does not only serve to help us understand biology; mathematics is biology. We are not less original if our thoughts resemble our mental constructions, we are just consistent. It is within this loop, this unavoidable recursion—mathematics understanding the logic of the brain—that we will have an opportunity to test, as some conspire, whether among all the wonders evolution has come out with, the ultimate might be a brain good enough to avoid the risk of understanding itself.
